# Isoliquiritin Ameliorates Cisplatin-Induced Renal Proximal Tubular Cell Injury by Antagonizing Apoptosis, Oxidative Stress and Inflammation

**DOI:** 10.3389/fmed.2022.873739

**Published:** 2022-03-30

**Authors:** Zhiyin Pei, Meng Wu, Hanqing Yu, Guangfeng Long, Zhen Gui, Xiaonan Li, Hongbing Chen, Zhanjun Jia, Weiwei Xia

**Affiliations:** ^1^Department of Clinical Laboratory, Children’s Hospital of Nanjing Medical University, Nanjing, China; ^2^Department of Children Health Care, Children’s Hospital of Nanjing Medical University, Nanjing, China; ^3^Department of Nephrology, Children’s Hospital of Nanjing Medical University, Nanjing, China; ^4^Jiangsu Key Laboratory of Pediatrics, Nanjing Medical University, Nanjing, China; ^5^Nanjing Key Laboratory of Pediatrics, Children’s Hospital of Nanjing Medical University, Nanjing, China

**Keywords:** cisplatin, renal proximal tubular cells, apoptosis, oxidative stress, inflammation

## Abstract

Acute kidney injury (AKI) is a clinical syndrome characterized by morbidity, mortality, and cost. *Cis*-diamminedichloroplatinum (cisplatin) is a chemotherapeutic agent used to treat solid tumors and hematological malignancies, but its side effects, especially nephrotoxicity, limit its clinical application. Isoliquiritin (ISL), one of the major flavonoid glycoside compounds in licorice, has been reported to have anti-apoptotic, antioxidant, and anti-inflammatory activities. However, the effect and mechanism of ISL on cisplatin-induced renal proximal tubular cell injury remain unknown. In this study, mouse proximal tubular cells (mPTCs) and human proximal tubule epithelial cells (HK2) were administered increasing concentrations of ISL from 7.8125 to 250 μM. Moreover, mPTC and HK2 cells were pretreated with ISL for 6–8 h, followed by stimulation with cisplatin for 24 h. CCK-8 assay was performed to evaluate the cell viability. Apoptosis and reactive oxygen species (ROS) of cells were measured by using flow cytometer and western blotting. Our results showed that ISL had no obvious effect on cell viability. ISL decreased cisplatin-induced cell injury in a dose-dependent manner. ISL also protected against cisplatin-induced cell apoptosis. Meanwhile, the enhanced protein levels of Bax, cleaved caspase-3/caspase-3 ratio, levels of Pp-65/p-65, levels of IL-6, and the production of ROS induced by cisplatin were significantly attenuated by ISL treatment. Moreover, ISL markedly increased the protein levels of Bcl-2 and SOD2, which were reduced by cisplatin stimulation. These results showed that ISL ameliorated cisplatin-induced renal proximal tubular cell injury by antagonizing apoptosis, oxidative stress and inflammation.

## Introduction

Acute kidney injury (AKI) is a group of clinical syndromes characterized by an abrupt decline in glomerular filtration (1) with high in-hospital morbidity (10–15% of all hospitalizations, sometimes exceeding 50% of patients in an intensive care unit) (2), mortality, (3), and cost (4). However, no drugs have been reported to be specifically, consistently, and reproducibly effective for curing AKI (2). Thus, the establishment of pharmacotherapy for AKI is urgently needed.

Cellular toxicity of drugs such as *cis*-diamminedichloroplatinum (cisplatin) is considered one of the major causes of AKI (5). As a chemotherapeutic agent, cisplatin is used to treat solid tumors and hematological malignancies owing to its broad antineoplastic properties (6, 7). However, the severe adverse effects of cisplatin, resulting from its non-tissue specificity, limit its clinical application (8, 9). More than 90% of cisplatin is absorbed by the kidney, resulting the accumulation of cisplatin in the kidney; thus, nephrotoxicity is one of the most common side effects (10, 11). Cisplatin accumulates in renal proximal tubular cells, contributing to inflammatory response, injury, and cell death (11). Increasing evidence suggests that apoptosis, inflammation and oxidative stress are involved in cisplatin nephrotoxicity.

Isoliquiritin (ISL) is one of the major flavonoid glycoside compounds in licorice, and is one of the oldest and most common medicines (12). Evidence indicates that ISL can profoundly attenuate lipopolysaccharide (LPS) or chronic social defeat stress (CSDS)-induced depressive symptoms and CSDC-induced anxiety behavior by restoring the inflammatory response and decreasing pyroptosis-related neuronal cell death (13). Meanwhile, it was found that ISL regulated genes related to oxidative stress and apoptosis pathways in an ischemic stroke zebrafish model (14). In addition, a recent study reported that ISL was capable of reducing the levels of TNF-α and IL-6 in hepatocytes (15). These findings indicate that ISL has anti-apoptotic, anti-oxidative, and anti-inflammatory properties. Therefore, we hypothesized that ISL may alleviate cisplatin-induced AKI through its anti-apoptotic, antioxidant, and anti-inflammatory actions.

To define the role of ISL in a cisplatin-induced renal proximal tubular cell injury, the toxicity of ISL to mouse proximal tubular cells (mPTCs) and human renal endothelial cells (HK2) was examined by a Cell Counting Kit-8 (CCK-8) assay in a dose-response experiment, and its effect on the cell viability, apoptosis, oxidative stress and inflammatory factor of mPTCs or HK2 cells induced by cisplatin was evaluated. Here, we examined whether ISL is a protective drug against a cisplatin-induced renal proximal tubular cell injury. Our research aimed to provide a potential therapeutic direction for pharmacotherapy for AKI.

## Materials and Methods

### Reagents

ISL was purchased from Selleck Chemicals (Shanghai, China), and cisplatin was obtained from Sigma-Aldrich (St. Louis, MO, United States). Antibodies against cleaved caspase-3 and caspase-3 (19677-1-AP), Bax (50599-2-Ig), Bcl-2 (26593-1-AP), β-actin and horseradish peroxidase-conjugated goat anti-mouse antibody (SA00001-1) were purchased from Proteintech Group (Rosemont, IL, United States). Antibodies against SOD2, NF-κB (p-65) and Phospho-NF-κB (Pp-65) were purchased from Cell Signaling Technology (Beverly, MA, United States). Horseradish peroxidase-conjugated goat anti-rabbit antibody and the reactive oxygen species (ROS) assay kit were purchased from Beyotime Biotech (Nantong, China). An apoptosis detection kit was purchased from BD Biosciences (San Diego, CA, United States). The CCK-8 assay kit was purchased from ApexBio (MA, United States). N-Acetylcysteine (NAC) was purchased from MedChemExpress. All other reagents were of reagent grade or better, and deionized water was used for all the experiments.

### Cell Culture and Treatment

HK2 and mPTCs were obtained from the American Type Culture Collection (ATCC, Manassas, VA, United States), and cultured in Dulbecco’s modified Eagle medium (DMEM) and DMEM/F-12 medium (319-075-CL, Wisent, Canada), respectively. The medium was supplemented with 10% (v/v) fetal bovine serum (FBS) (FBSSA500-S, AusGeneX, Molendinar, Australia). All the cells were maintained in a humidified atmosphere of 5% CO_2_ at 37^°^C.

### Establishment of a Cisplatin-Induced Cell Injury Model

When grown to 70% confluence, the HK2 and mPTC cells were deprived of FBS and administered with ISL [dissolved in dimethyl sulfoxide (DMSO), diluted with serum-free medium] for 6–8 h. Then, cisplatin was used to stimulate mPTCs (5 μg/mL) and HK2 (10 μg/mL) cells for 24 h.

### Assessing the Toxicity of Isoliquiritin on Cells by the Cell Counting Kit-8 Assay

HK2 and mPTCs were seeded in 96-well plates. When the cells reached 70% confluence, they were treated with different concentrations (7.8125, 15.625, 31.25, 62.5, 125, and 250 μM) ISL in serum-free medium for 24 h. Then, the cell viability was analyzed using a CCK-8 assay kit: 10 μL CCK-8 reagent was added to 100 μL serum-free medium for per well and the plates were incubated for 1 h at 37°C in 5% CO_2_. Absorbance was detected at 450 nm using a Multiskan FC microplate reader (Thermo Fisher Scientific, Waltham, MA, United States).

### Quantitative Reverse Transcription Polymerase Chain Reaction

Total RNA was collected using TRIzol reagent (Takara, Tokyo, Japan) according to the manufacturer’s instructions. One microgram of RNA was reverse transcribed into complementary DNA (cDNA) using the PrimeScript RT Reagent Kit (Takara, Tokyo, Japan). A 2 × ChamQ SYBR qPCR Master Mix (Low ROX Premixed, Vazyme, Nanjing, China) was used to perform QRT-PCR amplification using a QuantStudio 3 Real-Time PCR System (Applied Biosystems, Foster City, CA, United States). The level of mRNA was analyzed using the ΔΔCT method. β-actin was used as an internal control. The IL-6 forward primer sequence was 5′-ACAAAGCCAGAGTCCTTCAGAGAG-3′, and the IL-6 reverse primer sequence was 5′-TTGGATGGTCTTGGTCCTTAGCCA-3′. The β-actin forward primer sequence was 5′-GGCTGTATTCCCCTCCATCG-3′, and the β-actin reverse primer sequence was 5′-CCAGTTGGTAACAATGCCATGT-3′.

### Western Blotting

The treated cells were lysed in radioimmunoprecipitation assay (RIPA) buffer supplemented with protease inhibitor cocktail (Roche, Basel, Switzerland) and phosphatase inhibitor cocktail (Beyotime Biotech, Nantong, China) for 30 min on ice. The lysed cells were centrifuged for 15 min at 12,000 rpm and 4°C. The cellular supernatants were collected, and the bicinchoninic acid (BCA) Protein Assay Kit (Beyotime Biotech, Nantong, China) was used to measure the protein concentration. Total protein (30 μg/lane) was loaded for western blotting analysis. Primary antibodies were used at a dilution of 1:1,000, and secondary antibodies were used at a 1:2,000 dilution. Protein signals were detected using an enhanced chemiluminescence detection system (Bio-Rad, Hercules, CA, United States). Image J (Wayne Rasband National Institutes of Health, United States) was used to analyze the chemiluminescence intensities of the protein bands.

### Apoptosis Assay

Both supernatants and cells were collected after treatment, centrifuged and washed twice with washing buffer. Then, the cells were stained with Annexin V- fluorescein isothiocyanate (FITC) and propidium iodide (PI) using an apoptosis detection kit (BD Biosciences, 556547, San Diego, CA, United States) according to the manufacturer’s instructions. The apoptosis assay was performed using a flow cytometer (Beckman Coulter, Inc., Bria, CA, United States) after incubation for 15 min in the dark. The results were analyzed using CytExpert version 2.0 software (Beckman Coulter, Inc., Bria, CA, United States).

### Measurement of Reactive Oxygen Species

After treatment, the supernatants were replaced with serum-free medium supplemented with dichlorofluorescin diacetate (DCFH-DA) at a dilution of 1:1,000 using a ROS detection kit (Beyotime Biotech, Nantong, China). After incubation for 30 min, the cells were rinsed twice with phosphate-buffered saline (PBS), collected, and ROS levels were detected using a flow cytometer (Beckman Coulter, Inc., Bria, CA, United States). NAC was used as the positive control. The results were analyzed using CytExpert version 2.0 software (Beckman Coulter, Inc., Bria, CA, United States).

### Statistical Analysis

The data are presented as the mean ± standard deviation. Statistical analyses were performed by analysis of variance (ANOVA) followed by Turkey’s test for multiple groups using GraphPad Prism 5 software. A value of *P*< 0.05 was considered as statistically significant.

## Results

### Isoliquiritin Ameliorated Cisplatin-Induced Injury in Mouse Proximal Tubular Cells

**FIGURE 1:**
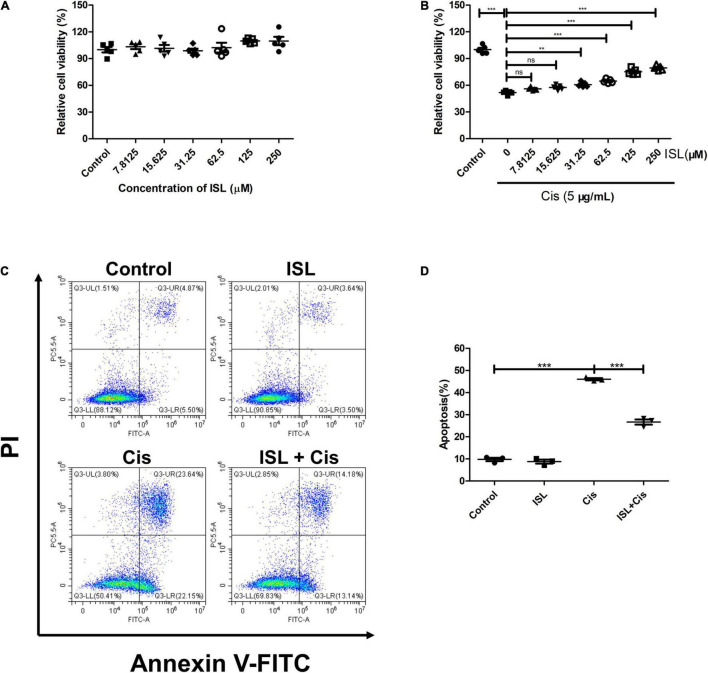
ISL protected mPTCs against cisplatin-induced injury without side-effects. **(A)** mPTCs were administered different concentrations of ISL from 7.8125 to 250 μM for 24 h. Cell viability was detected by a CCK-8 assay kit (*n* = 5 per group). **(B)** mPTCs were pretreated with different concentrations of ISL for 6–8 h followed by stimulation with 5 μg/mL cisplatin for 24 h. Cell viability was detected by a CCK-8 assay kit (*n* = 5 per group). **(C)** Representative flow cytometry analysis of Annexin V and PI staining. mPTCs were pretreated with 62.5 μM ISL for 6–8 h followed by stimulation with 5 μg/mL cisplatin for 24 h. **(D)** Quantification of flow cytometry (*n* = 3 per group). The data presented was expressed as mean ± *SD* of each group. **p < 0.01, ***p < 0.001 (ANOVA with multiple comparisons). Cis, Cisplatin; ISL, isoliquiritin; CCK-8, Cell Counting Kit-8; PI, propidium iodide; SD, standard deviation; ANOVA, analysis of variance.

To determine the effect of ISL on mPTCs, the cells were treated with increasing concentrations of ISL from 7.8125 to 250 μM for 24 h, and cell viability was examined using a CCK-8 assay kit. The results showed that ISL had no obvious effects on mPTC cell viability (Figure 1A), up to 250 μM. To determine the role of ISL in a cisplatin-induced cell injury, mPTCs were pretreated with increasing concentrations of ISL from 7.8125 to 250 μM for 6–8 h, followed by cisplatin stimulation for 24 h. CCK-8 assay results showed that ISL increased the viability of mPTCs, which was reduced by cisplatin treatment, at a dose of 31.25–250 μM (Figure 1B). Apoptosis is one of the main pathological features of cisplatin-induced nephrotoxicity. We designed an experiment to evaluate the effect of ISL on cisplatin-induced apoptosis. mPTCs were pretreated with 62.5 μM ISL for 6–8 h, followed by cisplatin stimulation for 24 h. We detected apoptotic cells by flow cytometry and found that ISL treatment remarkably decreased the percentage of apoptotic cells (Figures 1C,D). These results suggest that ISL attenuates cisplatin-induced mPTCs injury.

### Isoliquiritin Attenuated Cisplatin-Induced Mouse Proximal Tubular Cell Apoptosis by Regulating the Levels of Related Proteins

**FIGURE 2:**
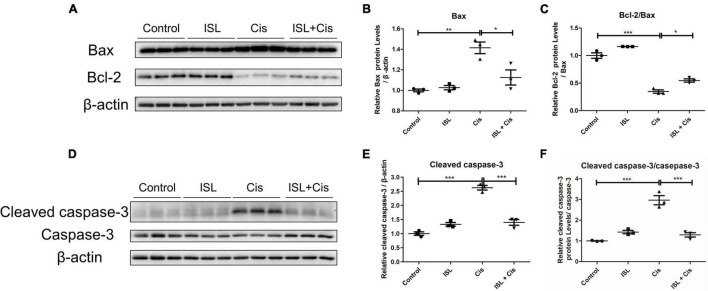
ISL alleviated cisplatin-induced apoptosis in mPTCs. mPTCs were pretreated with 62.5 μM ISL for 6–8 h and then incubated with 5 μg/mL cisplatin for 24 h. **(A)** The protein levels of Bax and Bcl-2 were detected by western blotting. β-actin was used as an internal reference. **(B,C)** Densitometry analysis of Bax **(B)** and Bcl-2/Bax **(C)**. **(D)** Protein levels of cleaved caspase-3 and caspase-3 were detected by western blotting. β-actin was used as an internal reference. **(E,F)** Densitometry analysis of cleaved caspase-3/β-actin **(E)** and cleaved caspase-3/caspase-3 **(F)**. The quantitative results are expressed as the mean ± *SD* of each group (*n* = 3 per group). *p < 0.05, **p < 0.01, ***p < 0.001 (ANOVA with multiple comparisons).

To further clarify the anti-apoptotic effect of ISL on mPTCs, the levels of apoptosis-related proteins such as Bax, Bcl-2, caspase-3, and cleaved caspase-3 were detected by immunoblotting. As shown in Figures 2A,B, the protein levels of Bax were significantly enhanced after cisplatin stimulation, however, its level was markedly blunted by ISL treatment. The protein levels of Bcl-2/Bax were significantly reduced after cisplatin stimulation, and were greatly increased by ISL treatment (Figures 2A–C). Consistent with the above results, the enhanced protein levels of cleaved caspase-3 and cleaved caspase-3/caspase-3 ratio in cisplatin-stimulated mPTCs were prominently decreased by ISL treatment (Figures 2D–F). All these results demonstrate a protective role of ISL against cisplatin-induced apoptosis in mPTCs.

### Isoliquiritin Alleviated Cisplatin-Induced Oxidative Stress and Inflammatory Response in Mouse Proximal Tubular Cells

**FIGURE 3:**
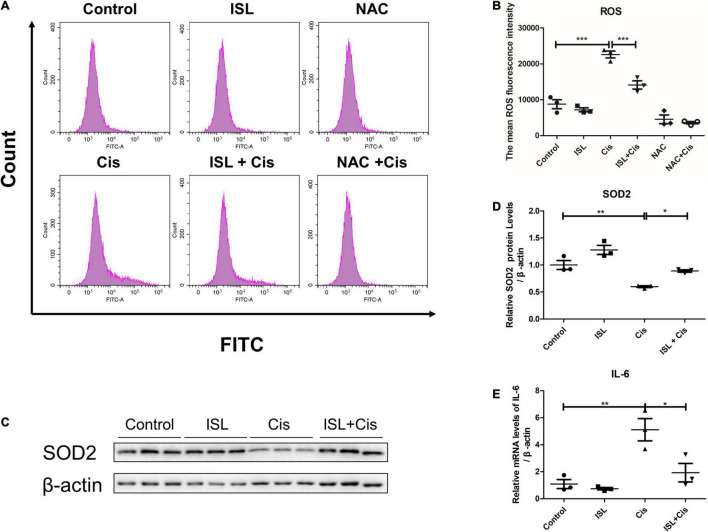
ISL suppressed cisplatin-induced oxidative stress and inflammation in mPTCs. **(A)** The representative images of the flow cytometry data of ROS assay in mPTCs. The mPTCs were pretreated with 62.5 μM ISL for 6–8 h, and then were administered 5 μg/mL cisplatin for 24 h. **(B)** Quantitation of the mean fluorescence intensity (*n* = 3 per group). **(C)** Representative western blots of SOD2 protein levels of mPTCs pretreated with 62.5 μM ISL for 6–8 h and administered 5 μg/mL cisplatin for 24 h. β-actin was used as an internal reference. **(D)** Densitometry analysis of the western blots of SOD2 (*n* = 3 per group). **(E)** The mRNA level of IL-6 was detected by QRT-PCR. The quantitative results are expressed as mean ± *SD* of each group. *p < 0.05, **p < 0.01, ***p < 0.001 (ANOVA with multiple comparisons).

Oxidative stress and inflammatory responses are also the main pathological features of cisplatin nephrotoxicity. Flow cytometry was performed to analyze the levels of total ROS in the mPTCs. The level of total ROS was apparently elevated after cisplatin stimulation; however, it was dramatically decreased by ISL treatment as shown in Figures 3A,B. Additionally, western blotting results showed that the protein expression of SOD2 was prominently downregulated after cisplatin stimulation compared to control group, and was significantly reversed by ISL treatment (Figures 3C,D). Furthermore, QRT-PCR detection of the cisplatin-induced inflammatory response showed that the greatly enhanced level of IL-6 following cisplatin stimulation was markedly decreased by ISL treatment (Figure 3E). All these findings indicate a potent role of ISL in alleviating cisplatin-induced oxidative stress and inflammatory response in mPTCs.

### Isoliquiritin Ameliorated Cisplatin-Induced Injury in HK2 Cells

**FIGURE 4:**
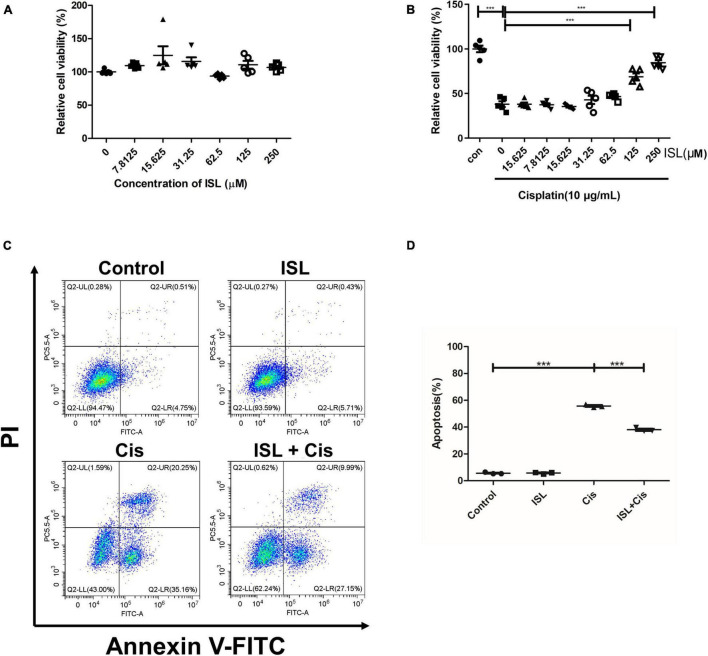
ISL protected HK2 cells against cisplatin-induced injury. **(A)** CCK-8 assay was performed to analysis the viability of HK2 cells administered increasing concentrations of ISL from 7.8125 to 250 μM for 24 h (*n* = 5 per group). **(B)** CCK-8 assay was performed to analysis the viability of HK2 cells pretreated with increasing concentrations of ISL from 7.8125 to 250 μM for 6–8 h followed by 10 μg/mL cisplatin stimulation for 24 h (*n* = 5 per group). **(C)** Representative flow cytometry analysis of Annexin V and PI staining. HK2 cells were pretreated with 125 μM ISL for 6–8 h followed by stimulation with 10 μg/mL cisplatin for 24 h. **(D)** Quantification of flow cytometry (*n* = 3 per group). The data presented is expressed as mean ± *SD* of each group. ***p < 0.001 (ANOVA with multiple comparison).

To further elucidate the protective effects of ISL against a cisplatin-induced human renal endothelial cell injury, we studied the effect of ISL on HK2 cells. HK2 cells were treated with increasing concentrations of ISL from 7.8125 to 250 μM for 24 h. CCK-8 assay was used to detect cell viability. As shown in Figure 4A, ISL treatment at a dose range of 0–250 μM did not cause any obvious toxicity. In addition, HK2 cells were treated with increasing concentrations of ISL from 7.8125 to 250 μM for 6–8 h and then treated with 10 μg/mL cisplatin for 24 h. The results showed that ISL treatment significantly increased cell viability, which decreased dramatically after cisplatin stimulation, when the concentration of ISL reached 125 μM (Figure 4B). Furthermore, HK2 cells were pretreated with 125 μM ISL for 6–8 h, followed by 10 μg/mL cisplatin stimulation for 24 h, and cell apoptosis was examined by flow cytometry. As shown in Figures 4C,D, ISL treatment significantly decreased the percentage of apoptotic cells induced by cisplatin in HK2 cells. These results demonstrate that ISL attenuate cisplatin-induced HK2 cells injury.

### Isoliquiritin Attenuated Cisplatin-Induced HK2 Cell Apoptosis by Regulating the Levels of Related Proteins

**FIGURE 5:**
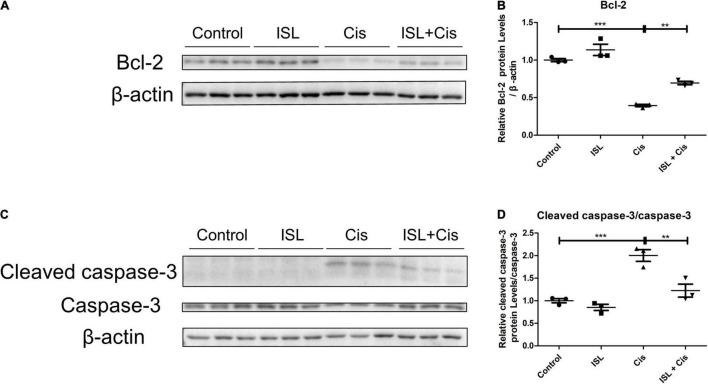
ISL reduced the cisplatin-induced apoptosis in HK2 cells. HK2 cells were pretreated with 125 μM ISL for 6–8 h and then incubated with 10 μg/mL cisplatin for 24 h. **(A)** The expression of Bcl-2 was detected by western blotting. β-actin was used as an internal reference. **(B)** Densitometry analysis of the western blots of Bcl-2 (*n* = 3 per group). **(C)** The expression of cleaved caspase-3 and caspase-3 was detected by western blotting. β-actin was used as an internal reference. **(D)** Densitometry analysis of cleaved caspase-3/caspase-3 (*n* = 3 per group). The quantitative results are expressed as the mean ± *SD* of each group. **p < 0.01, ***p < 0.001 (ANOVA with multiple comparison).

To determine the effect of ISL on cisplatin-induced change in apoptosis-related proteins, after pretreatment with 125 μM ISL for 6–8 h, HK2 cells were co-cultured with 10 μg/mL cisplatin for 24 h. Western blotting results showed that ISL distinctively increased the protein level of Bcl-2, which was decreased by cisplatin (Figures 5A,B). Consistent with the mPTC cell results, the protein level of cleaved caspase-3, which was notably enhanced by cisplatin stimulation, was reduced significantly by ISL treatment (Figures 5C,D). These results suggest a protective role of ISL against cisplatin-induced apoptosis in HK2 cells.

### Isoliquiritin Reduced Cisplatin-Induced Oxidative Stress and Inflammation in HK2 Cells

**FIGURE 6:**
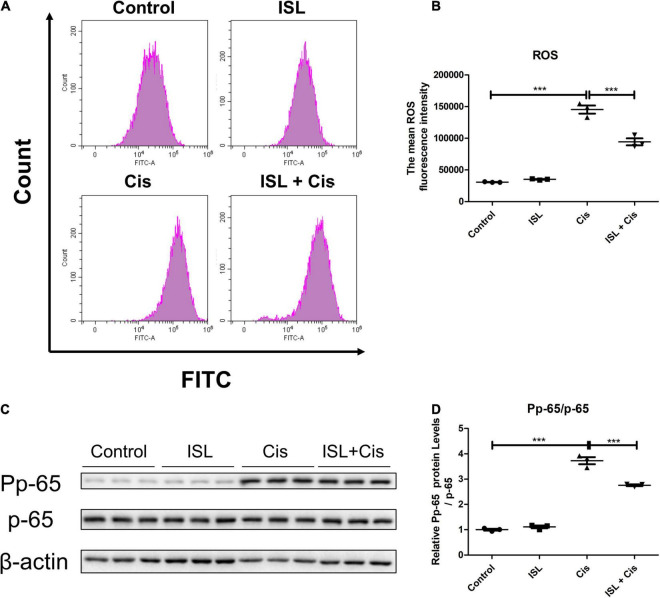
ISL ameliorated cisplatin-induced inflammation and oxidative stress in HK2 cells. **(A)** The representative images of the flow cytometry data of ROS assay in HK2 cells. The HK2 cells were pretreated with 125 μM ISL for 6–8 h, and then were administered 10 μg/mL cisplatin for 24 h. **(B)** Quantitation of the mean fluorescence intensity (*n* = 3 per group). **(C)** Representative western blots of Pp-65 and p-65 protein levels of HK2 cells pretreated with 125 μM ISL for 6–8 h and administered 10 μg/mL cisplatin for 24 h. β-actin was used as the internal reference. **(D)** Densitometry analysis of the western blots of Pp-65/p-65 (*n* = 3 per group). The quantitative results were expressed as mean ± *SD* of each group. ***p < 0.001 (ANOVA with multiple comparison).

Finally, flow cytometry was performed to examine ROS production. As shown in Figures 6A,B, the level of ROS was prominently elevated after cisplatin stimulation, which was apparently decreased by ISL treatment. In addition, western blotting results showed that the protein level of P p-65/p-65 was markedly enhanced by cisplatin, which was dramatically decreased by ISL treatment (Figures 6C,D). These results indicate that ISL alleviate cisplatin-induced oxidative stress and inflammatory responses in HK2 cells.

**FIGURE 7:**
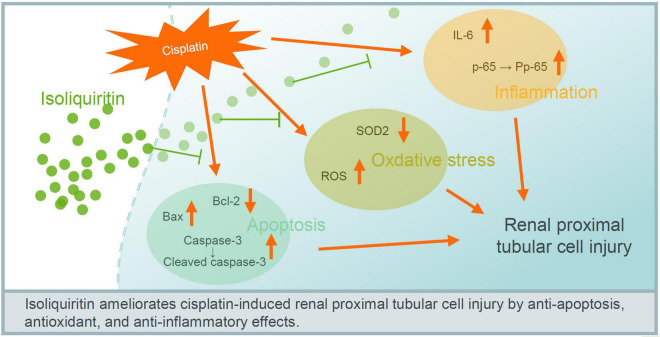
Proposed mechanism of ISL in protecting against cisplatin-induced renal tubular cell injury. ISL attenuate cisplatin-induced renal tubular epithelial cell injury possible via antagonizing apoptosis, oxidative stress, and inflammation.

Collectively, these results indicate that ISL attenuate cisplatin-induced renal tubular epithelial cell injury possibly via antagonizing apoptosis, oxidative stress, and inflammation (Figure 7).

## Discussion

Cisplatin has powerful anti-cancer properties, but its clinical application is extremely limited by its side effects, especially nephrotoxicity (11, 16). Combined with increasing incidence, high mortality, and high human and financial costs, AKI has become a major public health concern, with no effective therapeutic medicine currently available (2, 4).

Increasing evidences have shown that apoptosis played an important role in cisplatin-induced nephrotoxicity (17–20). There is a balance between the level of pro-apoptotic and anti-apoptotic proteins of B-cell lymphoma-2 family, which determines the fate of the cells to apoptotic stimulation. An excess of anti-apoptotic proteins such as Bcl-2 makes cells resilient, while an excess of pro-apoptotic proteins such as Bax makes cells sensitive to apoptosis. Once the balance exceeds the apoptotic threshold, the caspase cascade is activated, including caspase-3 (21–23). ISL is a major flavonoid glycoside and possess anti-apoptotic activities (12). In our study, we found that ISL significantly reduced the cisplatin-enhanced protein level of Bax in mPTCs and elevated the expression of Bcl-2, which was decreased by cisplatin in HK2 cells. The level of cleaved caspase-3, the active form of caspase-3, was greatly reduced by ISL, and was increased by cisplatin in both mPTCs and HK2 cells. Thus, ISL reduced cisplatin-induced apoptosis in renal tubular epithelial cells.

Oxidative stress has a crucial role in cisplatin-induced kidney injury (18, 24). The administration of cisplatin causes the accumulation of ROS, leading to cell damage including programmed cell death (25–27). In addition to the increase in ROS levels, the activity of antioxidants such as superoxide dismutase (SOD), which could limit ROS toxicity, is impaired after treatment with cisplatin (28, 29). SOD levels were markedly decreased after cisplatin treatment (20, 30, 31). Here, we observed that ISL dramatically decreased the level of ROS, which greatly increased after cisplatin administration in both mPTCs and HK2 cells. The protein expression of SOD2 was markedly elevated by ISL, which was significantly decreased by cisplatin in HK2 cells. This demonstrates that ISL dramatically decreased cisplatin-induced oxidative stress in renal tubular epithelial cells, which are consistent with those of previous studies (12, 32). These findings suggested that ISL not only decreased the production of ROS, but also could alleviate the ROS-induced programmed cell death to some degree. However, we also cannot rule out the potential of multiple mechanisms contributing to the protective role of ISL in the present experimental setting.

Inflammation also plays a crucial role in cisplatin-induced kidney injury (33, 34). IL-6, a typical cytokine for sustaining homeostasis, has a pathological effect on acute systemic inflammation once IL-6 level is excessively dysregulated and persistently synthesized (35, 36). Previous studies have showed that IL-6 levels are markedly elevated in cisplatin-induced nephrotoxicity (37, 38). A previous study showed that IL-6 was present predominantly in renal tubular epithelial cells rather than infiltrating neutrophils. Our study showed that ISL reduced the cisplatin-induced increase in IL-6 levels in mPTCs. This was consistent with the results of a recent study (13, 15). p-65 is a subunit of the nuclear factor kappa light chain enhancer of activated B cells (NF-κB), which is a transcriptional activator of inflammatory mediators (39). The protein level of Pp-65/p-65 was greatly enhanced after cisplatin treatment, as reported in the literature (40, 41). Here, we found that ISL decreased the protein level of Pp-65/p-65, which was elevated by cisplatin in HK2 cells. This demonstrates that ISL alleviates the inflammatory response induced by cisplatin in renal tubular epithelial cells.

Above all, some studies such as other renal injury model tests, animal studies, deeper mechanistic research and clinical tests are still needed to gain a deeper understanding of the protective activity of ISL in kidney injury. At present, we have discovered that ISL had a renal protective activity through anti-apoptosis, antioxidant, and anti-inflammatory pathways, which may open a new direction for improving the outcomes of AKI.

## Conclusion

We demonstrated for the first time that ISL protects the kidney against cisplatin-induced injury by regulating the apoptosis, inflammation, and oxidative stress-related pathways. Although deeper mechanistic studies, further animal tests, and clinical studies are still needed, our study provides evidence of the clinical potential of ISL in treating AKI.

## Data Availability Statement

The original contributions presented in the study are included in the article/supplementary material, further inquiries can be directed to the corresponding author/s.

## Author Contributions

WX, ZJ, and HC designed the experiments, analyzed the data, prepared the figures, and wrote the manuscript. ZP, MW, HY, GL, and ZG performed the experiments. XL contributed to technical advices. All authors reviewed the manuscript.

## Conflict of Interest

The authors declare that the research was conducted in the absence of any commercial or financial relationships that could be construed as a potential conflict of interest.

## Publisher’s Note

All claims expressed in this article are solely those of the authors and do not necessarily represent those of their affiliated organizations, or those of the publisher, the editors and the reviewers. Any product that may be evaluated in this article, or claim that may be made by its manufacturer, is not guaranteed or endorsed by the publisher.
